# Tree nut, peanut, and peanut butter intake and risk of postmenopausal breast cancer: The Netherlands Cohort Study

**DOI:** 10.1007/s10552-017-0979-7

**Published:** 2017-11-22

**Authors:** Piet A. van den Brandt, Lisette Nieuwenhuis

**Affiliations:** 1 Department of Epidemiology, Maastricht University Medical Centre, Care and Public Health Research Institute (CAPHRI), Maastricht, The Netherlands; 2 Department of Epidemiology, GROW- School for Oncology and Developmental Biology, Maastricht University Medical Centre, PO Box 616, 6200 MD Maastricht, The Netherlands

**Keywords:** Breast cancer, Nuts, Peanuts, Peanut butter, Cohort study

## Abstract

**Purpose:**

Nut intake has been associated with reduced mortality and risk of cardiovascular diseases, but there is only limited evidence on cancer. We investigated the relationship between nut intake and risk of postmenopausal breast cancer, and estrogen/progesterone receptor (ER/PR) subtypes.

**Methods:**

In The Netherlands Cohort Study, 62,573 women aged 55–69 years provided information on dietary and lifestyle habits in 1986. After 20.3 years of follow-up, 2,321 incident breast cancer cases and 1,665 subcohort members were eligible for multivariate case-cohort analyses.

**Results:**

Total nut intake was significantly inversely related to ER negative (ER −) breast cancer risk, with HR 0.55 (95% CI 0.33–0.93) for those consuming at least 10 g nuts/day versus non-consumers (*p* trend = 0.025). There were no significant inverse associations with ER + or total breast cancer. While there was no variation between PR subtypes, the ER–PR- subtype was also significantly inversely associated with nut intake, with HR 0.53 (95% CI 0.29–0.99), *p* trend = 0.037. Intake of peanuts and tree nuts separately was also inversely related to ER − breast cancer subtypes, while no associations were found with peanut butter intake.

**Conclusions:**

Our findings suggest an inverse association between nut intake and ER − breast cancer, and no association with total or hormone receptor-positive subtypes.

**Electronic supplementary material:**

The online version of this article (10.1007/s10552-017-0979-7) contains supplementary material, which is available to authorized users.

## Introduction

Nut intake has been associated with reduced risk of non-communicable diseases such as cardiovascular diseases (CVD) and diabetes [1]. Apart from CVD, interest is growing in mortality and other health effects as well, stimulated by the PREDIMED trial showing effects of Mediterranean diet supplemented with mixed nuts or olive oil on CVD and depression [2]. In several cohort studies, nut intake has been associated with lower total mortality and cancer mortality, e.g., [3–5], but few studies have been done on nut intake and risk of cancer. Also, little is known on differences between tree nuts and peanuts, and whether peanut butter shows similar associations with risk as peanuts. In addition, dose–response relationships remain unclear.

For breast cancer, two cohort studies have investigated the association between nut intake and breast cancer risk [6, 7]; both found no association with overall breast cancer risk. However, no distinction was made between tree nuts, peanuts, and peanut butter (these were grouped). One recent large population-based case–control study showed a significant inverse association between total nut intake in adolescent years and breast cancer (OR 0.76), with a stronger association for postmenopausal than premenopausal breast cancer [8]. Recent evidence from a randomized controlled trial on primary prevention of cardiovascular diseases indicated a potentially strong protective effect of Mediterranean diet supplemented with nuts on the risk of postmenopausal breast cancer in Spain; however, this was not significant, probably due to small number of cases during short follow-up [9]. For proliferative benign breast disease, a cohort study showed that two or more servings of nuts per week during adolescence were inversely associated with 36% lower risk of benign breast disease, compared with an intake of less than one serving per month. Statistically significant inverse associations were also observed for peanut intake alone [10].

It is important to distinguish between pre- and postmenopausal breast cancer, as well as estrogen/progesterone receptor (ER/PR) subtypes, because of differences in etiology.

We investigated the association between intakes of total nuts, tree nuts, peanuts, and peanut butter, and postmenopausal breast cancer risk, overall and stratified by hormone receptor status, in The Netherlands Cohort Study (NLCS). We recently found an inverse association between Mediterranean Diet (MD)-adherence and ER − breast cancer in the NLCS [11], in which nuts seemed to play a dominant role. Here, we further investigated this and evaluated tree nuts, peanuts, and peanut butter separately, while controlling for MD adherence.

## Materials and methods

### Study design and cancer follow-up

The NLCS started in September 1986 and the female part included 62,573 women aged 55–69 years [12]. At baseline, participants completed a mailed, self-administered questionnaire on cancer risk factors. The NLCS study was approved by institutional review boards from Maastricht University and The Netherlands Organization for Applied Scientific Research. All cohort members consented to participation by completing the questionnaire. For data processing and analysis, the case-cohort method was used [13]. Accumulated person-years in the cohort were estimated from a subcohort (*n* = 2589 women), randomly sampled from the cohort immediately after baseline. These subcohort members were actively followed up biennially for vital status information. The follow-up of the subcohort was 100% complete at 20.3 years of follow-up.

Follow-up for cancer incidence in the entire cohort was established by annual record linkage with The Netherlands Cancer Registry and PALGA, the nationwide Dutch Pathology Registry [14]. Completeness of follow-up through record linkage with cancer registries and PALGA was estimated to be greater than 95% [15]. After 20.3 years of follow-up (17 September 1986, until 1 January 2007), a total of 3,354 incident breast cancer cases (of whom 144 were also subcohort member) were detected among women. Cases and subcohort members were excluded if they reported a history of cancer (except skin cancer) at baseline and if their dietary data were incomplete or inconsistent [16]. Figure S1 (Supplementary data) shows the selection and exclusion steps that resulted in the number of cases and female subcohort members that were included in the analysis. There were 1665 subcohort members and 2,321 breast cancer cases available for analysis. As with the nested case–control study, the case-cohort study is also nested within a cohort with comparable efficiency gain. An additional advantage of the case-cohort design over the nested case–control design is that the selected subcohort can be used to study a range of disease endpoints [17].

### Exposure assessment

The 11-page baseline questionnaire measured dietary intake, detailed smoking habits, anthropometry, physical activity, and other risk factors related to cancer [12]. Habitual consumption of food and beverages during the year preceding baseline was assessed using a 150-item semi-quantitative food-frequency questionnaire, which was validated against a 9-day diet record [16]. Nut and peanut butter consumption was assessed by asking frequency and portion size of intake of ‘peanuts,’ ‘other nuts, mixed nuts,’ and ‘peanut butter.’ Frequency of consumption could range from ‘never or less than 1x/month’ to ‘6–7x/week.’ In addition, participants could fill in the number of standard portion sizes they consumed per intake. For tree nuts and peanuts, a standard portion size was 28 g. A standard portion size of peanut butter, a particularly popular spread in The Netherlands, was 15 g per slice of bread. Consumption frequencies and portion sizes were multiplied to calculate mean daily intakes in grams. Total nut intake was calculated as the sum of peanuts and other nuts. Nutrient intakes were calculated using the computerized Dutch food composition table [18].

### Statistical analysis

For the intakes of nuts and peanut butter, the mean (SD) values were calculated in the subcohort. The distribution of the subcohort members by nut intake level and various characteristics was examined by cross-tabulations and summary statistics.

The relationship between intake of nuts and breast cancer risk was evaluated using Cox proportional hazards models. It was verified that the proportional hazards assumption was not violated using Schoenfeld residuals [19] and − ln(− ln) survival plots. Standard errors were estimated using the robust Hubert–White sandwich estimator to account for additional variance introduced by the subcohort sampling [20].

In age- and multivariable-adjusted survival analyses, total nut intake was evaluated and tested on categorical (0, 0.1–< 5, 5–< 10, 10 + g/day) and continuous scales. In multivariable analyses, hazard ratios (HRs) were corrected for potential confounders: age at baseline (55–59, 60–64, 65–69 years), cigarette smoking status (never, former, current), frequency (number of cigarettes per day; continuous, centered), duration (number of years; continuous, centered), body height (continuous, cm), BMI (< 18.5, 18.5–< 25, 25–< 30, ≥ 30 kg/m^2^), non-occupational physical activity (≤ 30, > 30–60, > 60–90, > 90 min/day), highest level of education (primary school or lower vocational, secondary or medium vocational, and higher vocational or university), family history of breast cancer in mother or sisters (no, yes), history of benign breast disease (no, yes), age at menarche (≤ 12, 13–14, 15–16, ≥ 17 years), parity (nulliparous, 1–2, ≥ 3 children), age at first birth (< 25, ≥ 25 years), age at menopause (< 45, 45–49, 50–54, ≥ 55 years), oral contraceptive use (never, ever), postmenopausal hormone replacement therapy (never, ever), energy intake (continuous, kcal/day), alcohol intake (0, 0.1–< 5, 5–< 15, 15–< 30, ≥ 30 g/day). Because we recently found an association between Mediterranean diet adherence and breast cancer risk [11], we additionally adjusted for adherence to the Mediterranean diet as measured with the alternate Mediterranean diet (aMED) score [21]. Since nuts comprise one of the components of the aMED-score and because alcohol consumption is positively associated with breast cancer risk, an adapted version (excluding nuts and alcohol) was used here, which ranged from 0 (no adherence) to 7 (maximal adherence).

Linear trends between nut intake categories and breast cancer were evaluated with Wald tests, after fitting median values of nut consumption per intake category as continuous terms in the regression model. Median values were based on the distribution of the variables in the subcohort. Analyses were also done for peanuts and tree nuts separately, and peanut butter; because of lower numbers in the high intake categories, we used categories 0, 0.1–< 5, 5 + g/day.

Besides overall postmenopausal breast cancer, we conducted these analyses for subtypes defined by hormone receptor status: ER+, ER −, PR+, PR −, ER + PR+, and ER–PR. Differences in associations with nut intake between breast cancer subtypes were tested using a heterogeneity test [22], in which the standard error for the observed difference in rate ratios was estimated using a bootstrapping method developed for the case-cohort design [23].

To further investigate the dose–response relations between nut consumption and breast cancer risk, restricted cubic splines with three fixed knots (0, 5, and 10 g/day) were used to graphically present the dose–response curves without making a priori assumptions about their shapes. Wald tests were performed to evaluate the linearity of these relationships.

To evaluate potential residual confounding by breast cancer risk factors, and effect modification, analyses of MD-scores and breast cancer were also conducted within strata of alcohol intake, BMI, physical activity, and adapted aMED-score. Interactions with these factors were tested using Wald tests and cross-product terms. In sensitivity analyses, we repeated analyses after excluding cancers (and person-years) occurring in the first 2 years of follow-up. Moreover, analyses of peanut butter consumption were repeated, restricted to respondents who had stated having had the same peanut butter intake during the 5 years before baseline. Unfortunately, these data were unavailable for total nut, tree nut, and peanut consumption.

Analyses were performed using Stata version 12; presented p values are two-sided, with p < 0.05 considered as statistically significant.

## Results

Mean total nut consumption (SD) in subcohort women was 4.3 (8.4) g/day; for tree nut, peanut, and peanut butter, these values were 1.0 (3.9), 3.3 (6.9), and 1.2 (3.6), respectively. Nut consumers were on average somewhat younger (Table 1), leaner, drank more alcohol, less often reported a positive family history of breast cancer, and were less often never smokers. They were higher educated, more often had a late age at birth of their first child, scored higher on the adapted aMED-score, and more often used oral contraceptives and postmenopausal HRT. Peanut butter intake was weakly positively associated with nut intake.

**Table 1:** Baseline characteristics [mean (SD), or percentage] according to category of total nut intake in subcohort women with complete dietary data, Netherlands Cohort Study

Characteristic	Total nut intake (g/day)				Peanut butter intake (g/day)		
	0 g/day	0.1–< 5	5–< 10	10 + g/day	0 g/day	0.1–< 5	5 + g/day
	(*n* = 821)	(*n* = 735)	(*n* = 225)	(*n* = 246)	(*n* = 1493)	(*n* = 351)	(*n* = 183)
Age (years)	62.2 (4.4)	61.1 (4.2)	60.1 (4.0)	60.6 (3.9)	61.6 (4.3)	60.9 (4.0)	60.7 (4.0)
Height (cm)	164.9 (6.5)	165.5 (5.8)	165.6 (6.0)	165.8 (6.2)	165.4 (6.1)	165.1 (6.0)	165.2 (6.6)
BMI (kg/m^2^)	25.3 (3.8)	25.1 (3.4)	24.4 (3.1)	24.5 (3.3)	25.0 (3.6)	25.2 (3.4)	24.4 (3.2)
Physical activity (min/day)	62.9 (55.2)	65.8 (48.1)	71.7 (55.1)	60.8 (37.4)	62.3 (48.4)	72.8 (60.0)	68.3 (49.2)
Age at menarche (years)	13.7 (1.8)	13.6 (1.7)	13.7 (1.8)	13.6 (1.7)	13.6 (1.7)	13.7 (1.9)	13.8 (1.9)
Age at menopause (years)	48.7 (4.4)	48.7 (4.6)	49 (4.4)	48.9 (4.3)	48.7 (4.5)	48.6 (4.4)	49.6 (4.2)
Alcohol intake (g/day)	4.9 (9.5)	5.8 (8.8)	7.2 (9.3)	8.6 (10.9)	6.1 (9.7)	5.5 (9.1)	4.9 (7.9)
Peanut butter intake (g/day)	1.0 (3.5)	1.2 (3.7)	1.3 (3.5)	1.3 (3.3)			
Total nut intake (g/day)					4.1 (8.3)	5.2 (8.7)	4.9 (8.5)
Never smoker (%)	60.7	59.3	49.8	50.4	57.9	54.7	62.3
University or higher vocational education (%)	6.2	10.7	14.3	12.6	8.4	11.5	14.8
Nulliparous (%)	17.7	20.5	16.9	17.6	18.1	19.0	22.0
Age at first birth ≥ 30 years (% of parous)	22.8	19.5	26.6	26.7	22.4	21.3	25.9
Ever used oral contraceptives (%)	20.6	24.9	35.6	33.2	24.3	27.8	29.5
Ever used hormone replacement therapy (%)	12.2	13.8	13.3	16.4	13.8	13.5	9.7
Family history breast cancer (%)	8.4	10.2	6.7	7.7	9.1	9.1	5.5
History benign breast disease (%)	6.8	9.4	8.4	6.5	8.5	6.3	6.0
aMEDr^a^ score (excluding nuts) 5–7 pts (%)	20.5	27.3	26.7	32.1	23.4	27.9	33.3

^a^aMEDr: alternate Mediterranean Diet Score excluding alcohol

Peanut butter consumers were on average somewhat younger (Table 1), leaner, drank less alcohol, less often reported a history of benign breast disease or family history of breast cancer, and were more often never smokers, but were higher educated, more often had a late age at birth of first child, scored higher on the adapted aMED-score, and more often used oral contraceptives and less often postmenopausal HRT.

Table 2 shows results of age-adjusted and multivariable-adjusted analyses of the associations of total nut intake with total breast cancer risk, and risk of estrogen and progesterone receptor subtypes. Total nut intake was not associated with total breast cancer risk in categorical or continuous analyses. Compared to non-consumers of nuts, the HR (95% CI) of breast cancer for those consuming at least 10 g nuts/day was 0.91 (0.72–1.14) (*p* trend = 0.625) in multivariable analyses. Additional analyses adjusting for peanut butter intake yielded similar results (data not shown). ER + breast cancer also showed no associations, but ER − breast cancer was significantly inversely associated with total nut intake. Compared to non-consumers of nuts, the HRs (95% CIs) of ER − breast cancer for those consuming 0.1–< 5, 5–<10, or at least 10 g nuts/day, were 0.78 (0.56–1.08), 0.60 (0.34–1.05), and 0.55 (0.33–0.93), respectively (*p* trend = 0.025). No clear associations were seen for PR subtypes. Risk of ER–PR − breast cancer was significantly inversely related to total nut intake in multivariable-adjusted analyses with a HR for those consuming at least 10 g nuts/day versus non-consumers of 0.53 (95% CI 0.29–0.99) with *p* trend = 0.037. The ER + PR + subtype showed no significant association with total nut intake (Table 2). Heterogeneity tests across subtypes using bootstrapping were not significant (data not shown). Analyses for the ER+/PR − subtype closely resembled those of ER + subtypes and was therefore not reported; the number of ER −/PR + cases was too small for separate analyses.

**Table 2:** Hazard Ratio of breast cancer and subtypes, according to total nut intake in multivariable-adjusted^a^ analyses, Netherlands Cohort Study

	Total nut intake (g/day) (median)						
	0 g/day	0.1–< 5 g/day	5–< 10 g/day	10 + g/day	*p* trend	Continuous	*p* non-linear
	(0)	(2.1)	(7.8)	(15.7)		per 10 g/day	
Total breast cancer							
No. of cases	935	844	251	291		2,321	
Person-years in subcohort	11,322	10,646	3,067	3,897		28,932	
Age-adjusted HR	1	0.97	1.02	0.91	0.471	1.01	
(95% CI)		(0.83–1.12)	(0.81–1.27)	(0.74–1.12)		(0.94–1.09)	
Multivariable-adjusted HR	1	0.94	1.05	0.91	0.625	1.02	0.343
(95% CI)		(0.80–1.10)	(0.82–1.34)	(0.72–1.14)		(0.94–1.11)	
ER + breast cancer							
No. of cases	436	415	119	151		1121	
Age-adjusted HR	1	1.03	1.04	1.01	0.978	1.03	
(95% CI)		(0.86–1.23)	(0.79–1.36)	(0.79–1.29)		(0.95–1.13)	
Multivariable-adjusted HR	1	1.02	1.10	1.07	0.589	1.06	0.429
(95% CI)		(0.84–1.24)	(0.82–1.48)	(0.81–1.41)		(0.96–1.17)	
ER − breast cancer							
No. of cases	117	87	20	24		248	
Age-adjusted HR	1	0.79	0.64	0.58	0.022	0.84	
(95% CI)		(0.58–1.07)	(0.38–1.07)	(0.36–0.93)		(0.66–1.06)	
Multivariable-adjusted HR	1	0.78	0.60	0.55	0.025	0.83	0.054
(95% CI)		(0.56–1.08)	(0.34–1.05)	(0.33–0.93)		(0.64–1.07)	
PR + breast cancer							
No. of cases	273	277	69	84		703	
Age-adjusted HR	1	1.10	0.97	0.89	0.305	0.99	
(95% CI)		(0.90–1.35)	(0.70–1.33)	(0.67–1.20)		(0.89–1.10)	
Multivariable-adjusted HR	1	1.06	0.97	0.94	0.576	1.01	0.278
(95% CI)		(0.85–1.33)	(0.68–1.40)	(0.68–1.30)		(0.89–1.15)	
PR- breast cancer							
No. of cases	158	128	40	49		375	
Age-adjusted HR	1	0.85	0.92	0.86	0.557	1.02	
(95% CI)		(0.66–1.11)	(0.62–1.36)	(0.60–1.24)		(0.89–1.17)	
Multivariable-adjusted HR	1	0.89	0.95	0.87	0.637	1.02	0.240
(95% CI)		(0.67–1.18)	(0.62–1.47)	(0.58–1.31)		(0.88–1.19)	
ER + PR + breast cancer							
No. of cases	263	272	68	82		685	
Age-adjusted HR	1	1.12	0.99	0.91	0.351	0.99	
(95% CI)		(0.91–1.38)	(0.72–1.37)	(0.67–1.22)		(0.89–1.11)	
Multivariable-adjusted HR	1	1.08	1.00	0.95	0.596	1.01	0.344
(95% CI)		(0.86–1.36)	(0.69–1.43)	(0.68–1.31)		(0.89–1.15)	
ER −PR − breast cancer							
No. of cases	76	63	14	17		170	
Age-adjusted HR	1	0.86	0.65	0.61	0.064	0.89	
(95% CI)		(0.60–1.24)	(0.35–1.21)	(0.35–1.07)		(0.68–1.16)	
Multivariable-adjusted HR	1	0.86	0.61	0.53	0.037	0.85	0.100
(95% CI)		(0.58–1.27)	(0.31–1.18)	(0.29–0.99)		(0.64–1.14)	

^a^Multivariable analyses were adjusted for the following: age at baseline (55–59, 60–64, 65–69 years), cigarette smoking (status (never, former, current), frequency (number of cigarettes per day; continuous, centered), duration (number of years; continuous, centered)), body height (continuous, cm), BMI (< 18.5, 18.5–< 25, 25–< 30, ≥ 30 kg/m^2^), non-occupational physical activity (≤ 30, > 30–60, > 60–90, > 90 min/day), highest level of education (primary school or lower vocational, secondary or medium vocational, and higher vocational or university), family history of breast cancer in mother or sisters (no, yes), history of benign breast disease (no, yes), age at menarche (< 12, 13–14, 15–16, ≥ 17 years), parity (nulliparous, 1–2, ≥ 3 children), age at first birth (< 25, ≥ 25 years), age at menopause (< 45, 45–49, 50–54, ≥ 55 years), oral contraceptive use (never, ever), postmenopausal HRT (never, ever), energy intake (continuous, kcal/day), alcohol intake (0, 0.1–< 5, 5–< 15, 15–< 30, ≥ 30 g/day), alternate Mediterranean Diet Score excluding alcohol and nuts (0–2, 3–4, 5–7 pts)

Table 3 shows results of multivariable analyses for intake of peanut, tree nuts, and peanut butter separately. For peanuts and tree nuts, the pattern of associations resembled that for total nut intake, i.e., inverse associations were observed for ER − and ER–PR − subtypes, albeit non-significant. For peanuts, the HR (95% CI) for ER − breast cancer, comparing for 5 + versus 0 g /day was 0.63 (0.40–1.01), with *p* trend = 0.059. For tree nuts, this was 0.47 (0.21–1.09) for the same contrast (*p* trend = 0.079). Similarly, for ER–PR − breast cancer the HR (95% CI) was 0.46 (0.17–1.23), (*p* trend = 0.124), when comparing for 5 + versus 0 g tree nuts/day. Peanut butter intake was not associated with total breast cancer risk or its subtypes (Table 3). Excluding of the first 2 years of follow-up, or limiting the peanut butter analyses to those with stable intake over the past 5 years, did not materially change the results (data not shown).

**Table 3:** Hazard Ratio of breast cancer and subtypes, according to intake of peanuts, tree nuts and peanut butter, multivariable-adjusted^a^ analyses, Netherlands Cohort Study

	Intake of peanuts (g/day) (median)					Tree nuts (g/day) (median)					Peanut butter (g/day) (median)				
	0 g/day	0.1–< 5 g/day	5–< 10 g/day	*p* trend	Continuous	0 g/day	0.1–< 5 g/day	5–< 10 g/day	*p* trend	Continuous	0 g/day	0.1–< 5 g/day	5–< 10 g/day	*p* trend	Continuous
	(0)	(2.0)	(10.7)		per 10 g/day	(0)	(1.6)	(8.9)		per 10 g/day	(0)	(1.2)	(5.3)		per 10 g/day
Total breast cancer															
No. of cases	1087	855	379		2,321	1,649	566	106		2,321	1,702	416	203		2,321
Person-years in subcohort	13,457	10,600	4,875		28,932	20,351	6,880	1,701		28,932	20,932	5,245	2,754		28,932
Age-adjusted HR	1	1.01	0.97	0.771	1.04	1	1.02	0.77	0.114	0.94	1	0.98	0.90	0.364	0.86
(95% CI)		(0.87–1.16)	(0.81–1.17)		(0.95–1.14)		(0.88–1.19)	(0.58–1.03)		(0.80–1.11)		(0.83–1.16)	(0.72–1.13)		(0.71–1.05)
Multivariable-adjusted HR	1	1.00	0.98	0.809	1.04	1	1.00	0.80	0.191	0.95	1	1.05	1.01	0.965	0.95
(95% CI)		(0.86–1.17)	(0.79–1.20)		(0.94–1.14)		(0.84–1.18)	(0.58–1.10)		(0.79–1.16)		(0.88–1.26)	(0.79–1.29)		(0.77–1.16)
ER + breast cancer															
No. of cases	526	408	187		1,121	784	286	51		1,121	827	200	94		1,121
Age-adjusted HR	1	1.00	0.99	0.949	1.06	1	1.08	0.78	0.269	0.97	1	0.97	0.85	0.270	0.84
(95% CI)		(0.84–1.19)	(0.79–1.24)		(0.95–1.18)		(0.90–1.30)	(0.55–1.12)		(0.81–1.17)		(0.79–1.20)	(0.65–1.13)		(0.65–1.07)
Multivariable-adjusted HR	1	1.01	1.02	0.860	1.07	1	1.11	0.88	0.661	1.03	1	1.04	0.98	0.921	0.93
(95% CI)		(0.84–1.22)	(0.80–1.32)		(0.95–1.21)		(0.91–1.36)	(0.60–1.30)		(0.85–1.25)		(0.84–1.30)	(0.73–1.33)		(0.73–1.20)
ER − breast cancer															
No. of cases	129	88	31		248	181	60	7		248	183	40	25		248
Age-adjusted HR	1	0.86	0.65	0.055	0.86	1	0.99	0.46	0.055	0.62	1	0.86	1.00	0.996	0.92
(95% CI)		(0.64–1.16)	(0.43–1.00)		(0.67–1.11)		(0.72–1.36)	(0.21–1.02)		(0.29–1.29)		(0.60–1.25)	(0.63–1.58)		(0.62–1.37)
Multivariable-adjusted HR	1	0.87	0.63	0.059	0.86	1	0.94	0.47	0.079	0.60	1	0.90	1.10	0.721	0.97
(95% CI)		(0.63–1.21)	(0.40–1.01)		(0.67–1.11)		(0.65–1.34)	(0.21–1.09)		(0.26–1.36)		(0.61–1.33)	(0.66–1.82)		(0.64–1.48)
PR + breast cancer															
No. of cases	331	267	105		703	495	178	30		703	518	125	60		703
Age-adjusted HR	1	1.05	0.89	0.337	1.01	1	1.07	0.73	0.203	0.93	1	0.98	0.87	0.398	0.86
(95% CI)		(0.86–1.28)	(0.68–1.16)		(0.88–1.16)		(0.87–1.33)	(0.47–1.11)		(0.71–1.20)		(0.77–1.24)	(0.63–1.20)		(0.65–1.14)
Multivariable-adjusted HR	1	1.05	0.91	0.477	1.02	1	1.05	0.82	0.480	0.98	1	1.04	1.02	0.901	0.98
(95% CI)		(0.84–1.31)	(0.67–1.22)		(0.89–1.18)		(0.83–1.34)	(0.52–1.31)		(0.74–1.30)		(0.81–1.35)	(0.72–1.45)		(0.73–1.30)
PR- breast cancer															
No. of cases	182	128	65		375	262	96	17		375	279	66	30		375
Age-adjusted HR	1	0.87	0.95	0.851	1.05	1	1.08	0.76	0.400	0.91	1	0.92	0.78	0.236	0.75
(95% CI)		(0.68–1.13)	(0.69–1.32)		(0.90–1.23)		(0.83–1.41)	(0.45–1.31)		(0.67–1.24)		(0.68–1.25)	(0.51–1.18)		(0.51–1.12)
Multivariable-adjusted HR	1	0.91	0.94	0.782	1.04	1	1.13	0.83	0.623	0.94	1	0.95	0.82	0.399	0.78
(95% CI)		(0.69–1.20)	(0.64–1.36)		(0.88–1.23)		(0.84–1.52)	(0.47–1.47)		(0.69–1.29)		(0.69–1.31)	(0.52–1.29)		(0.52–1.17)
ER + PR + breast cancer															
No. of cases	321	261	103		685	481	175	29		685	500	125	60		685
Age-adjusted HR	1	1.06	0.90	0.387	1.01	1	1.09	0.72	0.210	0.93	1	1.01	0.90	0.537	0.88
(95% CI)		(0.87–1.30)	(0.69–1.18)		(0.88–1.16)		(0.88–1.34)	(0.47–1.12)		(0.72–1.21)		(0.80–1.29)	(0.65–1.25)		(0.67–1.17)
Multivariable-adjusted HR	1	1.06	0.91	0.505	1.02	1	1.06	0.81	0.458	0.98	1	1.08	1.06	0.750	1.00
(95% CI)		(0.85–1.32)	(0.68–1.24)		(0.88–1.18)		(0.83–1.35)	(0.51–1.30)		(0.74–1.30)		(0.84–1.40)	(0.74–1.51)		(0.75–1.34)
ER–PR − breast cancer															
No. of cases	86	61	23		170	122	43	5		170	122	29	19		170
Age-adjusted HR	1	0.87	0.70	0.167	0.92	1	1.04	0.48	0.124	0.69	1	0.92	1.11	0.687	1.07
(95% CI)		(0.61–1.24)	(0.43–1.14)		(0.70–1.21)		(0.72–1.51)	(0.19–1.20)		(0.30–1.60)		(0.60–1.41)	(0.66–1.87)		(0.70–1.62)
Multivariable-adjusted HR	1	0.88	0.63	0.105	0.88	1	0.98	0.46	0.124	0.65	1	0.99	1.20	0.537	1.14
(95% CI)		(0.60–1.28)	(0.36–1.09)		(0.66–1.17)		(0.63–1.51)	(0.17–1.23)		(0.25–1.66)		(0.63–1.56)	(0.67–2.14)		(0.72–1.80)

^a^Multivariable analyses were adjusted for the following: age at baseline (55–59, 60–64, 65–69 years), cigarette smoking [status (never, former, current), frequency (number of cigarettes per day; continuous, centered), duration (number of years; continuous, centered)], body height (continuous, cm), BMI (< 18.5, 18.5–< 25, 25–< 30, ≥ 30 kg/m^2^), non-occupational physical activity (≤ 30, > 30–60, > 60–90, > 90 min/day), highest level of education (primary school or lower vocational, secondary or medium vocational, and higher vocational or university), family history of breast cancer in mother or sisters (no, yes), history of benign breast disease

**Fig. 1:**
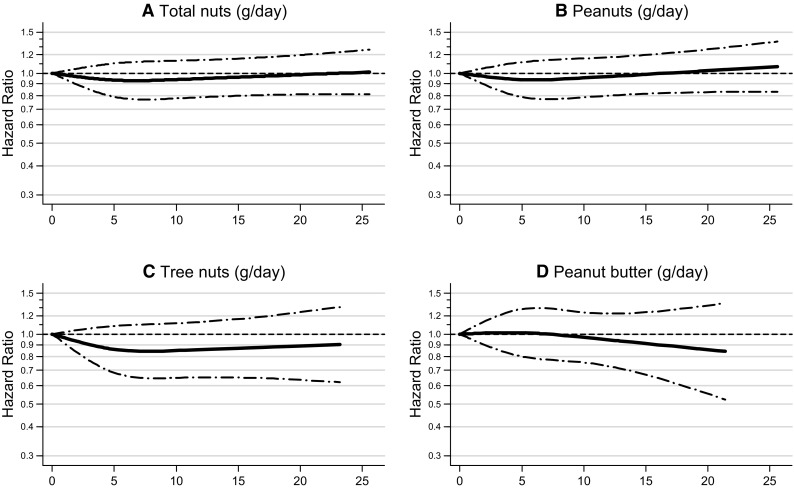
Non-parametric regression curves for the association between total breast cancer risk and **a** total nut intake, **b** peanuts, **c** tree nuts, and **d** peanut butter intake (g/day). Multivariate HRs are calculated by restricted cubic spline regression (using 3 knots at 0, 5, and 10 g/day) adjusting for the following: age at baseline (55–59, 60–64, 65–69 years), cigarette smoking [status (never, former, current), frequency (number of cigarettes per day; continuous, centered), duration (number of years; continuous, centered)], body height (continuous, cm), BMI (< 18.5, 18.5–< 25, 25–< 30, ≥ 30 kg/m^2^), non-occupational physical activity (≤ 30, > 30–60, > 60–90, > 90 min/day), highest level of education (primary school or lower vocational, secondary or medium vocational, and higher vocational or university), family history of breast cancer in mother or sisters (no, yes), history of benign breast disease (no, yes), age at menarche (≤ 12, 13–14, 15–16, ≥ 17 years), parity (nulliparous, 1–2, ≥ 3 children), age at first birth (< 25, ≥ 25 years), age at menopause (< 45, 45–49, 50–54, ≥ 55 years), oral contraceptive use (never, ever), postmenopausal hormone replacement therapy (never, ever), energy intake (continuous, kcal/day), alcohol intake (0, 0.1–< 5, 5–< 15, 15–< 30, ≥ 30 g/day), alternate Mediterranean Diet Score excluding alcohol and nuts (0–2, 3–4, 5–7 pts). To test for non-linearity, the model including the linear and cubic spline terms was compared to the model with only the linear term using a Wald test. *P* values for non-linearity were 0.343 for total nut intake, 0.347 for peanut intake, 0.212 for tree nuts and 0.683 for peanut butter intake. Lines with dashes represent the 95% confidence intervals (CIs) for the fitted non-linear trend (solid line)

**Fig. 2:**
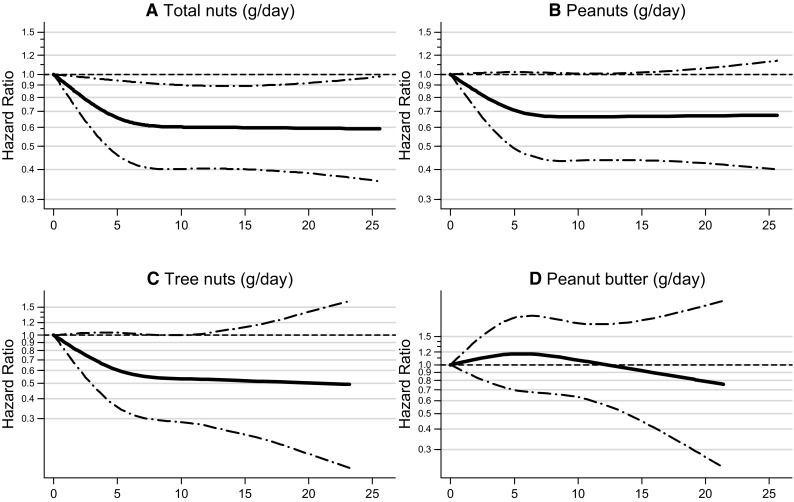
Non-parametric regression curves for the association between ER − breast cancer risk and **a** total nut intake, **b** peanuts, **c** tree nuts, and **d** peanut butter intake (grams/day). Multivariate HRs are calculated by restricted cubic spline regression (using 3 knots at 0, 5, and 10 g/day) adjusting for: age at baseline (55–59, 60–64, 65–69 years), cigarette smoking [status (never, former, current), frequency (number of cigarettes per day; continuous, centered), duration (number of years; continuous, centered)], body height (continuous, cm), BMI (< 18.5, 18.5–< 25, 25–< 30, ≥ 30 kg/m^2^), non-occupational physical activity (≤ 30, > 30–60, > 60–90, > 90 min/day), highest level of education (primary school or lower vocational, secondary or medium vocational, and higher vocational or university), family history of breast cancer in mother or sisters (no, yes), history of benign breast disease (no, yes), age at menarche (≤ 12, 13–14, 15–16, ≥ 17 years), parity (nulliparous, 1–2, ≥ 3 children), age at first birth (< 25, ≥ 25 years), age at menopause (< 45, 45–49, 50–54, ≥ 55 years), oral contraceptive use (never, ever), postmenopausal hormone replacement therapy (never, ever), energy intake (continuous, kcal/day), alcohol intake (0, 0.1–< 5, 5–< 15, 15–< 30, ≥ 30 g/day), alternate Mediterranean Diet Score excluding alcohol and nuts (0–2, 3–4, 5–7 pts). To test for non-linearity, the model including the linear and cubic spline terms was compared to the model with only the linear term using a Wald test. *P* values for non-linearity were 0.054 for total nut intake, 0.099 for peanut intake, 0.202 for tree nuts, and 0.474 for peanut butter intake. Lines with dashes represent the 95% confidence intervals (CIs) for the fitted non-linear trend (solid line)

Restricted cubic spline curves for the HR according to intake of total nuts, peanuts, tree nuts, and peanut butter are shown in separate panels in Fig. 1 for total breast cancer, and in Fig. 2 for ER − breast cancer. None of the tests for non-linearity were statistically significant (*p* values are shown in Figure legends). However, for total nuts and ER − breast cancer, it was borderline significant, and the exposure–response curves for total nuts, peanuts, and tree nuts show a clear leveling off with intake levels above 10 g/day. Therefore, effect modification and subgroup analyses were conducted using a categorical variable for nut intake: 0, 0.1–< 5, and 5 + g/day (combined upper category was needed because of sample size).

**Fig. 3:**
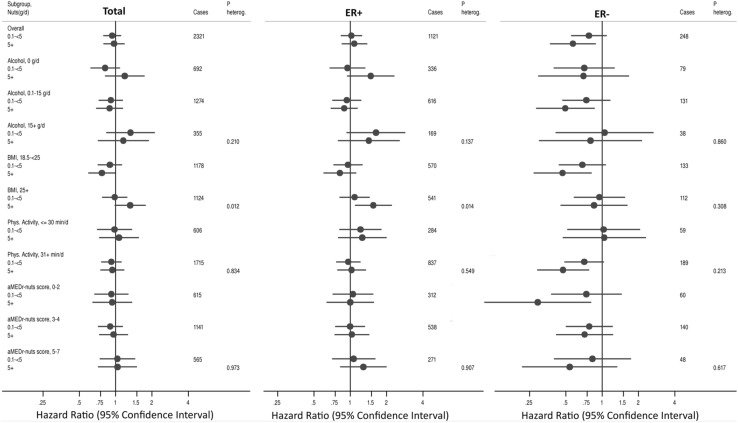
Hazard ratios and 95% confidence intervals of breast cancer, comparing total nut intake categories of 0.1–< 5, and 5 + g/day versus 0 g/day, in subgroups of potential effect modifiers. Multivariable analyses were adjusted for the following: age at baseline (55–59, 60–64, 65–69 years), cigarette smoking [status (never, former, current), frequency (number of cigarettes per day; continuous, centered), duration (number of years; continuous, centered)], body height (continuous, cm), BMI (< 18.5, 18.5–< 25, 25–< 30, ≥ 30 kg/m^2^), non-occupational physical activity (≤ 30, > 30–60, > 60–90, > 90 min/day), highest level of education (primary school or lower vocational, secondary or medium vocational, and higher vocational or university), family history of breast cancer in mother or sisters (no, yes), history of benign breast disease (no, yes), age at menarche (≤ 12, 13–14, 15–16, ≥ 17 years), parity (nulliparous, 1–2, ≥ 3 children), age at first birth (< 25, ≥ 25 years), age at menopause (< 45, 45–49, 50–54, ≥ 55 years), oral contraceptive use (never, ever), postmenopausal hormone replacement therapy (never, ever), energy intake (continuous, kcal/day), alcohol intake (0, 0.1–< 5, 5–< 15, 15–< 30, ≥ 30 g/day), alternate Mediterranean Diet Score excluding alcohol and nuts (0–2, 3–4, 5–7 pts)

In Fig. 3, HRs and 95% CIs for breast cancer and ER subtypes are presented for the two categories of total nut intake (0.1–< 5 and 5 + g/day) versus 0 g/day, overall, and in subgroups of potential effect modifiers: alcohol intake, BMI, physical activity, and adapted aMED-score (excluding alcohol and nuts). For total breast cancer, no association with nut intake was seen in most subgroups. For total and ER + breast cancer, there was significant interaction between nut intake and BMI-level, with decreased HRs observed in the subgroup BMI 18.5–< 25 [Total breast cancer: HR 0.79 (0.61–1.02); ER+: HR 0.84 (0.61–1.15)], and (significantly) increased HRs observed for BMI ≥ 25 kg/m^2^ [Total: HR 1.32 (0.96–1.81); ER+: HR 1.50 (1.03–2.18)]. For the ER − subtype, inverse associations were seen in most subgroups, and there was no significant interaction with these covariables. Possible interactions with age at baseline (55–59, 60–64, 65–69 years), smoking status (never, ex, current), and family history of breast cancer (no, yes) were also investigated, but were all non-significant.

## Discussion

In this large prospective study, we found a statistically significant inverse association between nut intake and risk of estrogen receptor-negative postmenopausal breast cancer. There were no significant inverse associations with ER + or total breast cancer risk. When comparing those consuming 10 + g nuts/day to non-consumers, the HR for ER − breast cancer was 0.55, while it was 0.91 for total breast cancer and 1.07 for ER + breast cancer. While there was no variation between PR subtypes, the ER–PR − subtype was also significantly inversely associated with nut intake. There was no statistical evidence of non-linear dose–response relationships, but this non-linearity test was borderline significant for ER − breast cancer, where the exposure–response curves show a clear leveling off with intake levels above 10 g/day. Intake of peanuts and tree nuts separately was also inversely related to ER − breast cancer subtypes, while no associations were found with peanut butter intake. There was significant interaction between total nut intake and BMI for total breast cancer and ER + breast cancer risk.

The PREDIMED randomized controlled trial in Spain investigated whether following a Mediterranean diet supplemented with nuts compared to a control diet in which it was advised to decrease dietary fat reduced the risk of breast cancer [9]. After a median follow-up of 4.8 years with 35 incident breast cancer cases, they found a RR of 0.59 (95% CI 0.26–1.35). Although this association was not significant, the HR was rather low and power was probably insufficient given the low number of cases. Since diets were analyzed and no direct comparisons between consumption of individual food items in both groups were made, it is unclear what the effect of nut consumption alone would be.

In two cohort studies, the relationship between nut consumption and risk of breast cancer was studied. Sonestedt et al. [6] in Sweden did not find a relation between nut intake and risk of breast cancer (HR for median consumption of 6 g/day vs. non-consumers = 0.98, 95% CI 0.75–1.27). Also, no relation was found when stratifying on ER status [6]. In the Nurses’ Health Study II, no association was observed between the number of servings of peanuts, peanut butter, and other nuts per day in young adulthood and risk of breast cancer in both premenopausal and postmenopausal women [7].

Besides these cohort studies, six case–control studies on this topic were identified. A case–control study in Argentina found non-significantly increased breast cancer risks with higher nut (peanut and walnut) consumption when comparing cases to both hospital and neighborhood controls [24]. An Italian case–control study found a significantly inverse association between seed oil consumption, including peanut oil, and breast cancer risk in both pre- and postmenopausal women [25]. In Canada, a significantly inverse relation was found between consumption frequency of total nuts during adolescence and breast cancer risk, which was mainly observed for postmenopausal breast cancer [8]. In Mexico, risk of breast cancer was significantly inversely related to the consumption frequency of peanuts, walnuts, and almonds [26]. Studies in Iran and the Central African Republic showed positive associations between (ground)nut intake and breast cancer, but no confounder adjustment was made [27, 28].

Two cohort studies were performed on nut intake and risk of benign breast disease, both in the US. Su et al. [10] found a statistically significant decreased risk of proliferative BBD when consuming nuts more often as adolescent (RR for ≥ 2/week vs. <1/month = 0.64, 95% CI 0.48–0.85), but this relation was not found for peanut butter consumption [10]. An inverse association between nut consumption in adolescence and subsequent BBD risk was also observed by Berkey [29].

Thus, the literature is mixed with null results in two cohort studies and inverse and positive associations in case–control studies (which may be linked to storage conditions of nuts in developing countries), but very few studies have investigated hormone receptor subtypes.

We also found no association with total breast cancer, but did find an inverse association with ER − subtypes. This needs to be confirmed in other large cohort studies and trials, with analyses per receptor subtype. We did not have data on nut intake in adolescence or early adulthood, which might be etiologically relevant; future studies might also focus on that.

In the NLCS, we earlier reported inverse associations with MD adherence in the ER − subtypes [11]. In the current effect-modification analysis, inverse associations with ER − breast cancer were seen in every subgroup of aMED excluding nuts (and alcohol), with the strongest inverse association found in those who had the lowest MD adherence score. This suggests an independent association between ER − breast cancer risk and nuts, apart from other MD-components. Nuts are a rich source of nutrients and energy, for example mono- and polyunsaturated fatty acids, protein, fiber, vitamins (e.g., various B-vitamins and vitamin E), minerals (e.g., magnesium, selenium), antioxidants, and phytochemicals like phenolic compounds and phytosterols [30], although the concentrations can vary among the different sorts of nuts [31]. The potential mechanisms of action of these components of nuts in the prevention of cancer have been investigated, but not in great detail. Some of them are related to antioxidant activity, the regulation of cell differentiation and proliferation, the reduction of tumor initiation or promotion, the repair of DNA damage, anti-inflammatory responses, the regulation of immunological activity, the induction or inhibition of metabolic enzymes and hormonal mechanisms [32, 33].

Although nuts have a high fat content, they contain mainly monounsaturated (MUFA) or polyunsaturated fatty acids (PUFA), and are very low in saturated fat [34]. When comparing peanuts to walnuts, it can be concluded that both are good sources of magnesium, MUFA and PUFA, but that walnuts contain more alpha-linolenic acid; peanuts are richer in MUFA, protein, niacin, and potassium. The antioxidant capacity of walnuts is higher than peanuts or peanut butter [5]. Peanuts, grapes, and red wine are primary sources of resveratrol. Resveratrol (a stilbene) has been shown to induce apoptosis, inhibit cell invasion and angiogenesis and has been tested in in vivo models of breast, colorectal, liver, pancreatic, and prostate cancer. In addition, resveratrol and anacardic acid (a phenolic acid in cashews) seem to be able to counteract cancer-related epigenetic alterations [35]. Epidemiologic studies and the PREDIMED trial have suggested an inverse association between nut consumption and inflammation [36]. Finally, inositol polyphosphates (from peanuts) might be related to energy metabolism and cancer through the inhibition of the PI3K/Akt pathway [35].

The prospective design and high completeness of follow-up of the NLCS make information bias and selection bias unlikely. A potential weakness is the moderate proportion of breast cancer cases for whom ER/PR status was known. Breast cancer cases with known and unknown receptor status did not differ importantly according to baseline and tumor characteristics, making selection bias of the cases unlikely (data not shown). Although many possible confounders were taken into account, the possibility of confounding by unmeasured factors remains. The validation study of the food frequency questionnaire has shown that it performs relatively well [16], but measurement error may still have attenuated associations. The lack of possibilities to update dietary intake or other lifestyle data during follow-up may have resulted in some attenuated associations too.

In conclusion, our cohort study showed a statistically significant inverse association between total nut intake and risk of ER − breast cancer. There were no significant inverse associations with ER + or total breast cancer risk. While there was no variation between PR subtypes, the ER–PR − subtype was also significantly inversely associated with nut intake. For ER − breast cancer, the exposure–response curves using restricted cubic splines showed a clear leveling off with intake levels above 10 g/day, but the non-linearity test was not significant. Intake of peanuts and tree nuts separately was also inversely related to ER − breast cancer subtypes, while no associations were found with peanut butter intake.

## Electronic supplementary material

Below is the link to the electronic supplementary material.

Flow diagram of the number of subcohort members and cancer cases on which analyses are based, Netherlands Cohort Study. (DOCX 48 KB)
