# Supplementary material for: Tree nut, peanut, and peanut butter intake and risk of postmenopausal breast cancer: The Netherlands Cohort Study
Source: Cancer Causes Control. 2017 Nov 22;29(1):63–75. doi: 10.1007/s10552-017-0979-7 (PMC5752734; doi:10.1007/s10552-017-0979-7)
Supplement: Supplementary file 1 — Flow diagram of the number of subcohort members and cancer cases on which analyses are based, Netherlands Cohort Study. (DOCX 48 KB) [file 10552_2017_979_MOESM1_ESM.docx]

**Supplementary materials.**
